# Physical activity level and associated factors among adult HIV patients in Ethiopia

**DOI:** 10.1186/s12879-022-07120-z

**Published:** 2022-02-04

**Authors:** Yadessa Tegene, Selamawit Mengesha, Caroline van der Starre, Stephanie Lako, Alemayehu Toma, Mark Spigt

**Affiliations:** 1 School of Public Health, College of Medicine and Health Science, Hawassa University, Hawassa, Ethiopia; 2 School of Medicine, College of Medicine and Health Science, Hawassa University, Hawassa, Ethiopia; 3 School CAPHRI, Department of Family Medicine, Maastricht University, Maastricht, Netherlands; 4 General Practice Research Unit, Department of Community Medicine, UiT the Arctic University of Norway, Tromsö, Norway

**Keywords:** Physical activity, People living with HIV, South Ethiopia

## Abstract

**Background:**

People living with HIV, who take antiretroviral therapy (ART), often enjoy long and healthy lives, but this therapy has well known metabolic adverse effects. Physical activity is found to be an important factor in improving these physiological parameters. This study aimed to determine physical activity level and associated factors among HIV patients in Ethiopia.

**Methods:**

An institutional based cross sectional study was conducted from May to June 2019. We selected a total of 422 adult HIV patients, attending antiretroviral therapy clinics in three selected hospitals in Southern Ethiopia. Data were collected at routine care consultations by nine trained nurses using a pre-tested structured questionnaire. The level of physical activity was measured by the international physical activity questionnaire (IPAQ).

**Result:**

The mean age of participants was 38.7 ± 9.13 years. Of the participants, 68% were physically inactive, with a higher proportion of inactive women (74%) than men (61%) [(AOR = 1.64, 95% CI (1.07, 2.53)]. In addition, urban vs. rural residents [(AOR = 2.57, 95% CI (1.16, 5.72)] and patients who were on ART for ≥ 24 months [(AOR = 1.88, 95% CI (1.15, 3.08)] had higher odds of having a low physical activity level.

**Conclusion:**

Most people living with HIV and receiving ART have low physical activity levels. Especially female and urban living patients and those with longer treatment duration have low levels of physical activity. More insight is needed on the reasons for physical inactivity among HIV patients and physical activity programs for HIV patients in low-income countries need to be developed.

## Introduction

In 2018, the estimated number of people living with HIV (PLWH) was 37.9 million, and 62% of them had access to life-saving antiretroviral medicines [1]. PLWH who take antiretroviral therapy (ART) can enjoy long and healthy lives [2]. This improvement caused the life expectancy of HIV patients and transformed HIV infection from an acute to a chronic disease [3]. However, the toxic side-effects of ART, long-term infection with HIV, the increased predisposition to obesity, and visceral adiposity have made PLWH more vulnerable to develop comorbidities, such as cardiovascular disease [4, 5].

Past studies shown the benefit of physical activity in preventing and managing the adverse effects of ART [6]. Physical activity is any bodily movement produced by skeletal muscles that results in energy expenditure [7]. It improves physiological parameters such as cardiorespiratory fitness, muscular strength, waist circumference, insulin resistance, blood lipid profile, HIV-associated lipodystrophy, and systemic inflammation [4, 8–10]. Furthermore, it could also benefit the mental health status of HIV patients by improving depression status and reducing anxiety [11].

Globally, the physical activity status of HIV patients varies between countries, on average 32% fall into the low physical activity category, the moderate and the high category, each accounts for 33%, as defined by the International Physical Activity Questionnaire (IPAQ) [12]. According to a systematic review comparing physical activity correlates in HIV patients across 45 countries the largest percentage of HIV patients were inactive in South America (55%) and highly active in North America (49%) and low levels of physical activity are mainly associated with exposure to ART, presence of lipodystrophy, old age, and a lower CD4 count [13]. Higher levels of physical activity are predominantly associated with a high educational level [13].

There is limited information available on the levels and effects of physical activity on HIV/AIDS patients in Sub-Saharan Africa. A study conducted in Uganda, showed the positive effects of physical activity among HIV-infected people treated with ART, especially on metabolic and cardiovascular-related risks [14]. However, the literature indicates that despite the clear benefits of physical activity for PLWH, studies on engagement in adequate physical activity are still limited. The main sources of physical activity in Ethiopia are related to work and transport [15]. It is expected that PLWH have lower physical activity levels as compared to the general Ethiopian population, as PLWH might be less capable of performing physical activities.

Currently, alike other parts of sub Saharan countries, the accessibility of ART for Ethiopian HIV patients is increasing and it is well known that this therapy is associated with adverse effects. Engaging in regular physical activity is one way to prevent and/or treat this adverse effect. Therefore, this research aims to assess the status of physical activity and examines the factors associated with the level of physical activity among adult PLWH in Ethiopia. The information obtained from this study provides useful information to design strategies in improving physical activity status and prevention of risks associated with physical inactivity among adult HIV patients.

## Methods

### Study design, setting, participants and sampling

This study was conducted using a cross-sectional design in three selected hospitals, one comprehensive specialized hospital and two general hospitals from May to June 2019. Hawassa University Comprehensive Specialized Hospital (HUCSH), a tertiary level hospital, delivers specialized and referral services for general hospitals. The two general hospitals, Adare and Yirgalem, deliver secondary level health care, providing preventive and curative services that require diagnostic facility and therapeutic intervention [16]. HUCSH and Adare general hospitals are found in Hawassa town, the capital of the Sidama regional state, and the South Nation Nationality Peoples Region (SNNPR) of Ethiopia, which is located, 275 km south of Addis Ababa, the capital of Ethiopia. HUCSH, Adare, and Yirgalem general hospitals at the beginning of this study gave ART service for 2553, 1821, and 1476 HIV patients respectively.

The study sample was selected from adult HIV patients (18 years plus), enrolled in ART care and visiting the three selected hospitals during the study period. Pregnant and lactating women were excluded from the study. A single population proportion formula was used to calculate the sample size. So far, there was no similar study conducted in the area or elsewhere having the same status as in Ethiopia. Therefore, the estimated proportion of 50% was taken to have maximum sample size. A sample of 384 was obtained and by considering the 10% non-response rate, the final sample size became 422. Proportional allocation was used to determine the number of study units to be sampled from each facility. Based on this, 184, 131 and 107 study participants were selected from HUCSH, Adare, and Yirgalem general hospitals, respectively. Individual study participants were selected by random arrival at the ART clinic.

### Data collection methods and procedures

Data were collected through interview administered questionnaires, conducted at routine consultation by nine trained nurses. A structured questionnaire was used to collect information on socioeconomic and ART-related characteristics of the study participants. To collect data on the level of physical activity of the participants, we used the the short form IPAQ [17]. Since several examples of physical activity were not regular activities in Ethiopia, we replaced these by other physical activities with approximately the same Metabolic Equivalent of Task (MET) [18]. In the category of vigorous physical activity, fast bicycling was replaced by rope jumping. In the category of moderate physical activity, bicycling at a regular pace and double tennis was replaced by cleaning and gardening. After the cultural adaptation, the questionnaire was translated to Amharic and retranslated to the original version to check for consistency. We have presented the data on physical exercise as a continuous score using MET-min per week (MET level × minutes of activity × events per week) or as a categorical variable in three categories: low, moderate, and high. Participants who did not fulfill the criteria of moderate and high were considered as low active or inactive.

Participants who fulfilled one of the following criteria were categorized in the moderate group.

- Performing at least 20 min of vigorous activity on 3 or more days a week or;
- Performing moderate-intensity activity or walking for at least 30 min on 5 or more days a week or;
- Any combination of moderate-intensity activity, vigorous activity or walking on 5 or more days achieving at least 600 MET-min per week.

Participants were categorized into the high activity group, if they fulfilled one of the following criteria:

- Performing vigorous-intensity activity on a minimum of 3 days a week and achieving at least 1500 MET-min per week or;
- Any combination of moderate-intensity activity, vigorous activity or walking on 7 days achieving at least 3000 MET-min per week.

Dietary diversity and household food insecurity data were collected using the Food and Nutrition Technical Assistance (FANTA) indicator guide for Household Dietary Diversity Score (HDDS) [19] and Household Food Insecurity Access Scale (HFIAS) [20] respectively. Anthropometric measurements, such as height, was measured using a stadiometer (Seca Germany) by positioning the patient at the Frankfert plane recorded to the nearest 0.1 cm. Weight was measured using a pretested and calibrated digital Seca^®^ scale and recorded to the nearest 0.1 kg.

Blood pressure (BP) was measured with the standard mercury sphygmomanometer BP cuff with the appropriate cuff size by measuring the left arm consistently, three times at a 5-min interval. The average of the two last readings was taken, and the diagnosis of high BP (hypertension) was made according to the WHO criteria as systolic BP ≥ 140 mmHg or diastolic BP ≥ 90 mmHg [21]. Random blood glucose levels were determined by using Fia Biomed Blood Glucose Meter (Glucometer) Salut by finger puncture. According to the American Diabetes Association’s guideline, fasting plasma glucose levels ≥ 126 mg/dL, 2 h plasma glucose ≥ 200 mg/dL during oral glucose tolerance test, hemoglobin A1C ≥ 6.5% and in a patient with classic symptoms of hyperglycemia or hyperglycemic crisis a random plasma glucose ≥ 200 mg/dL are defind as diabetes [22]. Participants who were unaware of the fact that they had diabetes and/or hypertension were linked to the respective hospital for further diagnosis and management of their conditions.

### Data management and analysis

Data were analyzed using SPSS for windows version 20.0 (IBM, USA). Descriptive statistics were presented in the form of frequency, percentage, mean and standard deviation. Chi square test was used to assess the association between categorical variables. Variables having *P*-value < 0.25 in the bivariate logistic regression analyses were considered as potential candidates in the final multivariable logistic regression analysis. *P*-value < 0.05 was used to declare statistical significance in the multivariable model. The overall goodness of fit of the model was checked by using Hosmer–Lemeshow. Finally, the adjusted odds ratio (AOR) with its 95% confidence interval (CI) was used to determine statistical significance.

## Results

### Socio-demographic characteristics of adults living with HIV/AIDS

We included a sample of 422 adult HIV patients receiving ART service from three selected public hospitals. Most participants (64%) were women and from an urban area (93%), with a mean age of 38.7 ± 9.13 years. Nearly half of the participants (48%) were married and had a monthly household income of ≤ 1500 Ethiopian Birr (52%). The majority of participants (37%) had completed secondary education and about 28% were privately employed (Table 1).

**Table 1:** Socio-demographic characteristics of people living with HIV/AIDS attending ART clinic (n = 422)

Variables	Frequency	Percent
Age		
< 20	8	2
21–30	75	18
31–40	192	45
41–50	102	24
51–60	45	11
Gender		
Male	154	36
Female	268	64
Marital status		
Single	203	48
Married	65	15
Divorced	75	18
Widowed	79	19
Occupation		
Government employee	95	23
Private employee	118	28
Daily-laborer	49	12
House wife	9	2
Merchant	65	15
Others	86	20
Educational level		
No formal education	44	10
Primary education	114	27
Secondary education	155	37
Tertiary education	109	26
Place of residence		
Urban	394	93
Rural	28	7
Income level		
< *$*33.98	218	52
≥ *$*33.98	204	48

Private Employee = A person who works for a private employer or in private organization and receives regular remuneration in salary

1USD = 44.15 Ethiopian Birr, 2021

### Clinical and nutrition related characteristics of adult people living with HIV/AIDS

More than half of the participants (58%) had a recent CD4 count that was ≥ 500 cells/mm^3^ and most patients had normal BMI (59%). Majority of the participants (92.2%) were in clinical stage I, had been on treatment for ≥ 2 years (70%), had no chronic comorbidity (11%), and household food insecurity (75%) (Table 2).

**Table 2:** Clinical and nutrition related characteristics of people living with HIV/AIDS attending ART clinic (n = 422)

Variables	Frequency	Percent
CD4 count		
< 200	44	10
200–349	62	15
350–499	72	17
≥ 500	244	58
WHO clinical stage		
Stage I	389	92
Stage II	15	3
Stage III	16	4
Stage IV	2	1
Drug regimen		
AZT-3TC-EFV	68	16
AZT-3TC-NVP	80	19
TDF-3TC-EFV	217	51
Others	57	14
Duration of treatment in months		
< 24	126	30
≥ 24	296	70
Chronic comorbidity		
No	375	89
Yes	47	11
BMI		
Underweight	63	15
Normal	247	59
Overweight	112	26
HHFIS		
Secured	107	25
Unsecured	315	75

AZT: Zidovudine; EFV: Efavirenz; NVP: Nevirapine; TDF: Tenofovir; 3 TC: Lamivudine

### Physical activity levels

We observed significant relationship between gender and PA level (χ^2^ = 6.08, P ≤ 0.048), where men were, in general, more physically active than women. Participants living in rural areas were found to have more overall PA (P = 0.007) compared to other urban living study participants (Table 3).

**Table 3:** Domain-specific physical activity level among adults living with HIV/AIDS attending ART clinic, in three selected public hospitals of South Ethiopia, 2019, (n = 422)

Variables	Physical activity level						X^2^-P-value
	Low		moderate		Vigorous		
	N	%	N	%	N	%	
Gender							
Male	94	33	35	47	25	42	0.48^*^
Female	194	67	40	53	34	58	
CD4 count							
< 200	35	12	6	8.0	3	5	0.16
200–349	40	14	16	21	6	10	
350–499	47	16	10	13	15	25	
≥ 500	166	58	43	57	35	59	
Educational level							
No formal education	31	11	8	11	5	9	0.95
Primary education	79	27	18	24	17	29	
Secondary and above	178	62	49	65	37	63	
Place of residence							
Urban	275	96	64	85	55	93	0.007^*^
Rural	13	5	11	15	4	7	
BMI							
Under weight (≤ 18.5)	43	15	10	13	10	17	0.93
Normal (18.5–24.9)	166	58	47	63	34	58	
Over weight (≥ 25)	79	27	18	24	15	25	
Occupation							
Government employee	68	24	15	20	12	20	0.69
Private employee	83	29	15	20	20	34	
Daily-laborer	34	12	8	11	7	12	
House wife	6	2	2	3	1	2	
Merchant	39	14	16	21	10	17	
Others	58	20	19	25	9	15	

^*^P ≤ 0.05

### Physical activity level of the study participants

**Fig. 1:**
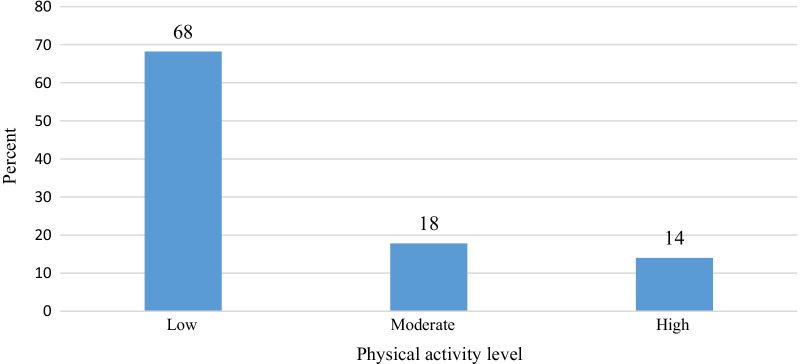
Physical activity levels of people living with HIV/AIDS attending ART clinic, in three selected public hospital of South Ethiopia, 2019, (n = 422)

**Fig. 2:**
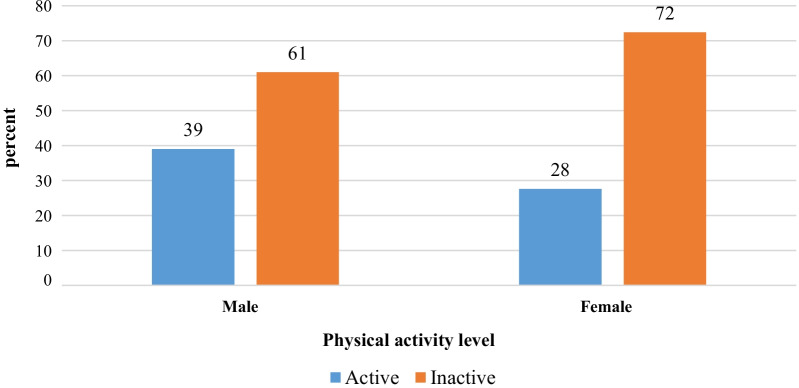
Gender stratified physical activity levels of people living with HIV/AIDS attending ART clinic, in three selected public hospital of South Ethiopia, 2019, (n = 422)

More than half of the participants, 288 (68%) had low PA levels, followed by moderate, 75 (18%) and high 59 (14%) respectively (Fig. 1). More, 194 (72%) women were physically inactive compared to men 94 (61%) (Fig. 2).

### Factors associated with physical activity level among adults living with HIV/AIDS

In bivariate logistic regression analysis; gender, place of residence, duration of treatment and HHFIS were factors associated with physical activity level. In multiple logistic regression analysis; gender, place of residence and duration of treatment remained significantly associated with physical activity level of participants. Women participants were two times [(AOR = 1.64, 95% CI (1.07, 2.53)] more likely to be physically inactive than men. Participants from urban residence were three times [(AOR = 2.57, 95% CI (1.16, 5.72)] more likely to be physically inactive than rural patients. Participants, who had been on ART for ≥ 24 months were nearly two times [(AOR = 1.88, 95% CI (1.15, 3.08)] more likely to be physically inactive than those who were on ART for < 24 months (Table 4).

**Table 4:** Factors associated with low levels of physical activity among adults living with HIV/AIDS attending ART clinic, in three selected public hospitals of South Ethiopia, 2019, (n = 422)

Variable	Low physical activity		COR (95% CI)	AOR (95% CI)
	No N (%)	Yes N (%)		
Gender				
Male	60 (39)	94 (61)	1	1
Female	74 (28)	194 (72)	1.67 (1.11–2.55)^*^	1.64 (1.07, 2.53)^*^
Place of residence				
Urban	119 (30)	275 (70)	2.67 (1.23, 5.78)^*^	2.57 (1.16, 5.72)^*^
Rural	15 (54)	13 (46)	1	1
Duration of treatment ( months)				
< 24	29 (23)	97 (77)	1	1
≥ 24	105 (35)	191 (65)	1.84 (1.14, 2.97)^*^	1.88 (1.15, 3.08)^*^
HHFIS				
Secured	29 (27)	78 (73)	1	1
Unsecured	105 (33)	210 (67)	1.35 (0.83, 2.19)	0.74 (0.45, 1.23)

^*^Statistically significant variables in multiple logistic regressions at P-value ≤ 0.05

## Discussion

We assessed the level of physical activity and the associated factors among HIV patients attending ART clinics of public hospitals in South Ethiopia. The current study showed a high proportion of physically inactive HIV patients, and women participants take the largest share of this proportion. Being an urban resident and a longer duration of treatment were also factors that were associated with physical inactivity.

In the current study, a high proportion (68%) of HIV patients attending the ART clinic was physically inactive. This result is consistent with study conducted in Malawi, which showed percentage of 40% [23]. These earlier studies indicated that being older was a leading reason for low PA among PLWHA [13]. In our study the majority of participants (89%) were younger aged (< 50 years) with the mean age of 38.7 years. In addition, we excluded pregnant and lactating women. Therefore, being pregnant did not explain the lower PA in the current study. Since the lower PA level was observed among younger and productive groups of the society, this poses a challenge in the economy of the country in addition to exposing them to an associated risk of chronic comorbidities. Therefore, physical activity interventions should get attention in the routine HIV management programs of the country.

In our study, a higher number of women were physically inactive. Consistent findings were seen in several studies conducted elsewhere in Africa [15, 23, 24]. In the current study area, men are involved more often in intensive manual labors, like agricultural activities, construction and often carrying heavy weights, than women. In addition, in the current study set up, women have less chance to engage in different recreational and regular physical exercise activities. Therefore, interventions aimed at promoting physical activity should target adult female HIV patients.

In the current study, participants residing in urban areas had higher odds of low PA than those in rural areas. This finding is in line with the studies conducted in sub-Saharan Africa countries, Northern Tanzania and Vietnam [25, 26]. In Ethiopia, people living in the rural area are more often involved in vigorous and moderate activities than urban people, because more manual labor and agricultural activities are found in the rural area than urban. In addition, most Ethiopian rural setups do not have transportation access, and due to that people walk long distances from one farm site to another or from place to place for various social issues. In addition, rural dwellers tend to practice manual work and active travel, which insures vigorous and moderate PA, while their urban counterparts seem to adopt sedentary lifestyles. Therefore, to avoid the additional burden of chronic comorbidity, it is vital to promote physical activity among urban PLWH as an intervention strategy.

The other variable that has shown a significant association with physical inactivity was the duration of treatment. Physical inactivity was more common among participants who stayed on ART for more than 2 years. Our finding is consistent with a study conducted in Vietnam [25]. The possible reason could be that patients may have more commitment at the initiation of treatment due to their health condition and the frequent counseling and follow-up they get from health care providers. Maybe, when their health status is stable in the later stages, they become less compliant to adhere to the care taken. Health care providers should consider developing peer support programs, regular follow-up and awareness creation to increase the level of physical activity and overall health status.

As a limitation, during our assessment of physical activity level, data were collected based on self-reported information, which is subjected to social desirability bias and results in over reporting of physical activity. Since participants were asked about their physical activity of 1-week duration, their response may also be subjected to recall bias. To minimize the bias, clear instruction was given on the objective, benefit and drawback of the study both for participants and data collectors.

## Conclusion

In our study most people living with HIV and receiving ART have low physical activity levels. Our study revealed the significant association between being female, urban residents and longer duration of treatment with physical inactivity. There is a need for physical activity programs for HIV patients.

## Data Availability

The datasets used and/or analyzed during the current study are available from the corresponding author on reasonable request.

## Abbreviations

AIDS: Acquired immune deficiency virus
AOR: Adjusted odds ratio
ART: Antiretroviral therapy
BP: Blood pressure
BMI: Body Mass Index
CI: Confidence interval
COR: Crude odds ratio
DM: Diabetes Mellitus
HHFIS: House Hold Food Insecurity Scale
HIV: Human immune virus
HUCSH: Hawassa University Comprehensive Specialized Hospital
IDDS: Individual Dietary Diversity Score
IPAQ: International Physical Activity Questionnaire
MET: Metabolic Equivalent of Task
NCD: Non-communicable disease
PLWH: People living with HIV
PA: Physical activity
SD: Standard deviation
SPSS: Statistical Package for the Social Sciences
WHO: World Health Organization
